# Antimicrobial Plants Used by Fang Populations and Phytochemical Profiling of *Erismadelphus exsul*

**DOI:** 10.3390/molecules29153503

**Published:** 2024-07-26

**Authors:** Morel Essono Mintsa, Cédric Sima Obiang, Elodie Choque, Elodie Dussert, Rozenn Ravallec, Joseph-Privat Ondo, Christophe Belloncle, Brice Serge Kumulungui, François Mesnard

**Affiliations:** 1 UMRt BioEcoAgro 1158-INRAE, BIOPI, Université de Picardie Jules Verne, 1 Rue des Louvels, 80000 Amiens, France; elodie.choque@u-picardie.fr; 2 Laboratoire Innovation Matériau Bois Habitat (LIMBHA), Ecole Supérieure du Bois, 7 Rue Christian Pauc, 44306 Nantes, France; christophe.belloncle@esb-campus.fr; 3 Laboratoire de Recherches en Biochimie, Université des Sciences et Techniques de Masuku, Franceville BP 943, Gabon; cedricsima@gmail.com (C.S.O.); wansbop70@yahoo.fr (J.-P.O.); 4 UMRt BioEcoAgro 1158-INRAE, Institut Charles Violette, Université de Lille, 59655 Lille, France; elodie.dussert@gmail.com (E.D.); rozenn.ravallec@univ-lille.fr (R.R.); 5 Centre Interdisciplinaire de Recherches Médicales de Franceville, Franceville BP 769, Gabon; kumulungui@yahoo.fr

**Keywords:** antibacterial activity, antioxidant activity, cytotoxicity, *Erismadelphus exsul*, ethnopharmacology surveys, Fang populations, mass spectrometry, molecular networking, traditional medicinal plant

## Abstract

Gabon has a rich flora, many species of which are used in traditional medicine. However, little research has been carried out on this wealth. An ethnopharmacological survey in the Fang language was carried out among traditional practitioners to collect antimicrobial medicinal plants. Phytochemical profiling of ethanolic and methanolic extracts from *Erismadelphus exsul* Mildbr leaves was carried out using HPLC-ESI-Q/TOF and a molecular network approach. Antibacterial activity was assessed with disk diffusion and microdilution methods, antioxidant activity via DPPH and FRAP methods, and in vitro cell viability via Cell Counting Kit-8. A total of 21 medicinal plants were collected, grouped into 10 families, of which the Fabaceae is the most represented. *Erismadelphus exsul* was chosen for chemical and biological studies due to its citation frequency (RCF = 0.59) and the absence of previous phytochemical studies. These studies revealed 4 major families of natural compounds and annotated 19 compounds for the first time. The crude leaf extract showed significant antioxidant and antibacterial activity. Cytotoxicity studies showed that the leaves were not cytotoxic, unlike the bark. This study underlines the importance of preserving the ancestral knowledge of the Fang populations, while showing promising results for *Erismadelphus exsul*.

## 1. Introduction

Traditional medicine makes a significant contribution to the global healthcare system, providing accessible and often culturally appropriate care to many populations. Its proper recognition is a key element of global health policy, as it enables the integration of proven practices and the valorization of ancestral knowledge, thus contributing to a more inclusive and holistic approach to healthcare [1]. Over the past decade, the use of traditional medicine has received renewed attention worldwide. For example, it accounts for a significant proportion of healthcare in China, Chile, Colombia, and India [2,3]. According to the World Health Organization (WHO), 80% of the world’s population treat their health problems with traditional remedies, due to the difficulties of access to modern medicine and the efficacy and lower toxicity of herbal treatments [4,5]. Recently, the WHO has called for the integration of traditional medicine into global healthcare systems [6]. A major challenge remains regarding the implementation of national healthcare systems that adequately develop traditional medicine policies and programs.

In Africa, and particularly in Gabon, medicinal plants play a crucial role in the treatment of various diseases, including those of microbial origin [7,8]. Gabon’s pharmacopoeia has significant potential, with a large proportion of the population using traditional medicine for primary health care [9]. Gabon is covered by primary tropical forests, offering exceptional plant potential that has yet to be fully explored [10]. The valorization of medicinal species is mainly based on ethnopharmacological surveys and phytochemical studies, through the analysis and identification of bioactive constituents. Recent advances in chemometrics, based on the processing and analysis of mass spectrometry data, have considerably improved the chemistry of living organisms [11]. In this context, liquid chromatography coupled to tandem mass spectrometry (HPLC-MS/MS) and the molecular network (MN) approach were used in this study to rapidly identify and target chemical components present in the leaves of *Erismadelphus exsul* Mildbr, a Vochysiaceae used in traditional Gabonese medicine to treat microbial diseases.

There is great interest in the search for new antimicrobial agents due to the resurgence of antibiotic-resistant infections. Essential oils and plant extracts have formed the basis of many pharmaceutical, medical, and agri-food applications. A significant proportion of the medicines currently available are derived from plants [12]. In 2011, the WHO called for intensified research into new drugs in the face of the dramatic increase in antibiotic resistance, but few new molecules are being developed. Plants synthesize a number of molecules, many with antibiotic and antioxidant activity, which can serve as reliable sources of new drugs that are relatively less toxic for humans and the environment [5].

This study focused on the selection and collection of plant species of therapeutic interest in the treatment of microbial diseases among the Fang populations of northern Gabon, with the aim of carrying out chemical and biological studies on plant species that have not been studied in the field of natural substances or for which there are insufficient data in the literature. We have thus assessed the antibacterial, antioxidant, and cytotoxic properties of the bark and leaves of *Erismadelphus exsul* Mildbr in view of its current use in traditional Gabonese medicine. The main priority remains the transfer of this scientific information to traditional practitioners, so that ethnopharmacological work can integrate traditional medicine into the health system where it does not exist. In this way, ethnopharmacology contributes to the advent of sustainable alternative solutions to the health problems of populations in emerging countries.

## 2. Results

One of the main aims of this study was to determine the ingredients used in the preparation of the remedies, to understand the methods of preparation, and to scientifically evaluate the use of these plants by the Fang populations of northern Gabon. A total of 20 traditional practitioners took part in the survey. All had already treated at least one and up to 40 patients suffering from diseases of microbial origin. The survey showed that men outnumbered women (60% versus 40%). The age range most represented among these traditional practitioners is between 40 and 50 (Table 1). In terms of education, more than half the healers have completed elementary school. According to their experience, healers were classified into three categories: those with more than 20 years’ experience (25%), those with less than 10 years’ experience (70%), and those with more than 30 years’ experience (5%) (Table 1). 

In order to obtain a certain homogeneity of information on the use of medicinal plant species in relation to certain disease categories, we determined informant consensus factors (ICFs). ICFs are important because they measure the degree of agreement between informants on the use of certain plants for specific ailments, helping to validate the reliability of the traditional knowledge collected. Higher ICF values indicate greater consensus and potentially greater reliability and importance of the medicinal uses of these plants. Diseases were classified into five main groups: infection-related diseases, skin-related diseases, digestive system-related diseases, fever- and cough-related diseases, and sterility-related diseases (Table 2). The results show that the ICFs range from 0.16 to 0.83. Indeed, diseases related to digestive problems have the lowest ICF (0.16), while diseases associated with skin problems have the highest (0.83).

I. Diseases associated with infections: urinary tract infections, pneumoniae, gastrointestinal disorder, diarrhea, chronic diarrhea, malaria, pulmonary infection, anti-poison, dysentery, mycosis, ringworms, gonorrhea, body and mouth odor, sexual weaknesses, and sexual infections;
II. Diseases associated with skin: eczema, wounds, and burns;
III. Diseases related to digestive problems: hemorrhoids, abdominal pain, constipation, ulcers, and stomach pain;
IV. Diseases associated with infertility: male infertility, female infertility, fallopian tube obstruction in women, and clogged trumpets;
V. Diseases associated with fever and coughs: bronchitis, cough, chronic cough, rheumatism, and fever.

### 2.1. Identified Plants: Parts Used and Method of Preparation of Recipes

**Figure 1:**
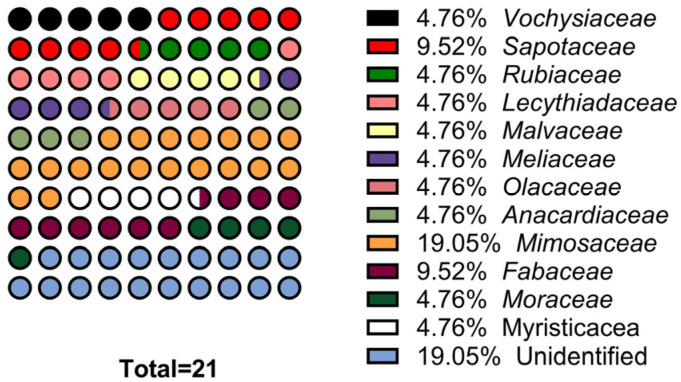
Ethnopharmacological surveys showing the frequency of botanical families studied.

The exploitation of the survey data allowed for the identification of 18 species used in traditional medicine by the Fang populations (northern ethnic group of Gabon) in the treatment of microbial diseases. A total of 21 plants were collected (Table 3), including 4 unidentified and 17 identified plants. These plant species were grouped into 10 families, of which the most representative families are, successively, the following: Fabaceae, Mimosaceae, and Sapotaceae (Figure 1). In general, bark represents the most used plant organ (59.10%), due to its ease of collection by village populations. Our results are in agreement with the work of Ngoua Meye et al. (2019), who showed in their ethnopharmacological study conducted in Northern Gabon that bark is the most used plant organ in traditional recipes [8]. Scientifically, the bark, fruits, and leaves are the best seat of the metabolites responsible for the curative properties of plants [13]. Decoction is the most widely used preparation method and remains the quickest way to extract the active ingredients [14,15]. In this study, decoction was used in 54.56% of cases versus 22.71% for maceration, with other preparation methods accounting for 22.73%. These results are in agreement with those obtained by Sima Obiang et al. (2023), on studies of aromatic medicinal plants used in northern Gabon in the locality of Oyem [16]. Water was used as an extraction solvent to prepare the recipes against microbial diseases. The oral route was the main method of administering the recipes, followed by sitz baths, with other routes being in the minority. The results obtained during the surveys enabled us to determine the main uses of the organs of several medicinal plants on the outskirts of the towns of Oyem and Bitam. Among the 21 plants collected, *Erismadelphus exsul* Mildbr was selected for phytochemical studies. In fact, the choice of this plant was inspired not only by information from the field but also because of the plant’s citation frequency (RCF = 0.59), far higher than that of the other plants in this study (Table 3). 

Indeed, RCF (citation frequency) plays a key role in identifying the medicinal plants most commonly cited and used by traditional practitioners. Quantifying RCF enables these plants to be prioritized for more detailed phytochemical studies, as high citation frequency suggests greater recognition and perceived efficacy. Thus, plants with a high RCF can be considered promising candidates for phytochemical studies. On the other hand, plants with low RCF may require further ethnobotanical exploration to understand the reasons for their limited use. In addition to the importance of RCF, the absence of pre-existing studies on *Erismadelphus exsul* reinforces the value of original research. To date, very little work has been done on this plant, with the notable exception of Essono Mintsa et al. (2022) [17], who, for the first time, carried out chemical and biological studies on *Erismadelphus exsul*. The combination of a high RCF and the absence of studies justifies the choice of this plant.

### 2.2. Molecular Networks of Erismadelphus exsul Leaf Extracts

**Figure 2:**
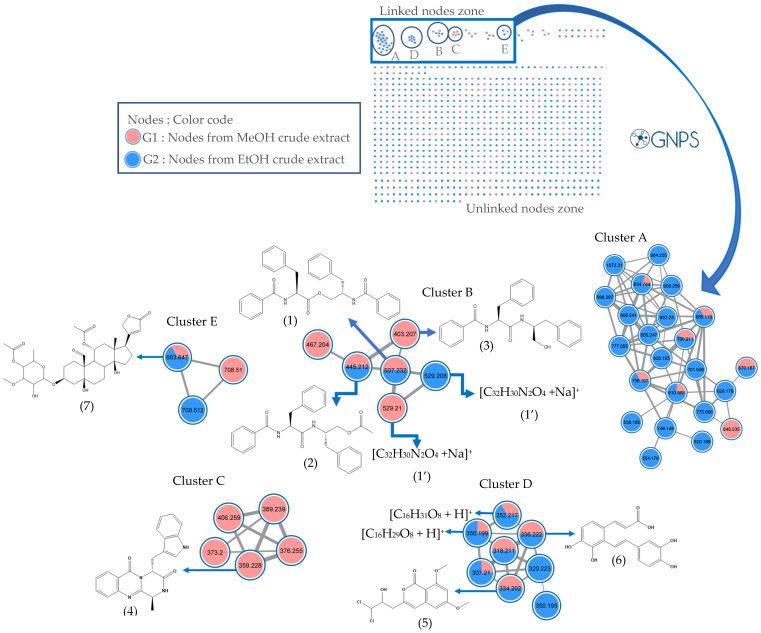

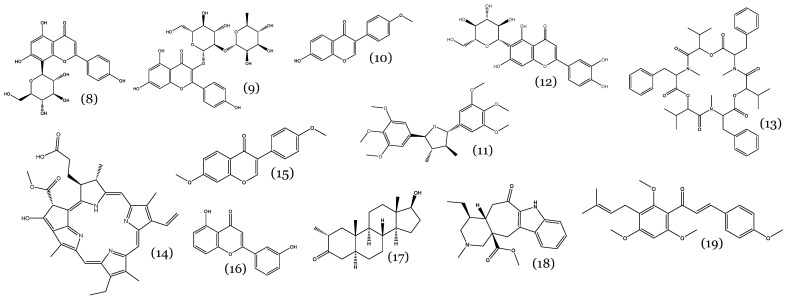
Molecular network analysis of *Erismadelphus exsul* crude ethanolic extract and leaf fractions obtained with the GNPS platform and visualized with Cytoscape 3.9.0 software. This molecular network shows compounds annotated on each cluster (compounds **1** to **7**) and compounds annotated at unlinked nodes called selfloops (compounds **8** to **19**). The names of the compounds identified are as follows: (1) Asperphenamate, (2) Asperglaucide, (3) Aurantiamide, (4) Fumiquinazoline F, (5) 3-(3,3-dichloro-2-hydroxypropyl)-6,8-dimethoxyisochromen-1-one, (6) salvianolic acid F, (7) Sarmentoside B, (8) Vitexin, (9) Kaempferol 3-neohesperidoside, (10) Formononetin, (11) Grandisin, (12) Isoorientin, (13) Beauvericin, (14) Pheophorbide A, (15) 4′, 7-Dimethoxyisoflavone, (16) 5,3′-Dihydroxyflavone, (17) Drostanolone, (18) Ervatamine, and (19) (*E*)-3-(4-methoxyphenyl)-1-[2,4,6-trimethoxy-3-(3-methylbut-2-enyl)phenyl]prop-2-en-1-one. Nodes in red represent nodes from the crude extract and those in blue represent nodes from the fractions.

The molecular network obtained from HPLC-MS^2^ data in positive ionization mode of methanolic and ethanolic extracts of *Erismadelphus exsul* Mildbr leaves was generated from 1770 individual MS^2^ spectra, organized into 1356 nodes, forming five clusters (zone of connected nodes) and several selfloops (zone of unlinked nodes). Among the five clusters, four clusters (B, C, D, and E) were of particular interest to us as they grouped related nodes facilitating not only the putative identification of several compounds but also the different families of compounds. The red nodes correspond to the MS^2^ spectra of the compounds present in the methanolic crude extract, and the blue nodes correspond to the MS^2^ spectra of the ethanolic crude extract (Figure 2). A total of 19 compounds were annotated in the leaf extracts of the plant *Erismadelphus exsul* Mildbr (Figure 2 and Table 4). Among the compounds identified, 12 appear in unlinked nodes called selfloops (Figure 2), and the remaining 7 appear in linked-node zones (clusters B, C, D, and E). Observation of clusters A and C showed that the nodes of cluster C correspond to compounds originating exclusively from the methanolic crude extract, while those of cluster A originate predominantly from the ethanolic crude extract. Comparison of the crude formulas of cluster C with databases such as DNP (Dictionary of Natural Products) and PubChem yielded no results except for the *m*/*z* 359.228 [C_21_H_18_N_4_O_2_ + H]^+^ node which corresponds to Fumiquinazoline F, an antitumor compound naturally produced by *Aspergillus fumigatus* [18] (Table 4). The lack of correspondence of these nodes in different databases suggests that they are potentially new compounds and good candidates for isolation. Cluster E analysis shows a single matching node *m*/*z* 663.64 [C_34_H_48_O_13_ + H]^+^, mainly from the ethanolic crude extract. This fragment corresponds to the MS^2^ spectrum of sarmentoside B, a steroid glycoside isolated from the plant *Strophanthus sarmentosus* [19,20]. However, further purification and identification of the compound is required to confirm its identity. Analysis of cluster B showed a node at *m*/*z* 445.212 [C_27_H_28_N_2_O_4_ + H]^+^, corresponding to the MS^2^ spectrum of asperglaucide. This compound matched in both types of extract (Figure 2). In this cluster, we also listed two other nodes corresponding to the MS^2^ spectrum of asperphenamate at *m*/*z* 507.232 [C_32_H_30_N_2_O_4_ + H]^+^ and aurantiamide *m*/*z* 403.207 [C_25_H_26_N_2_O_3_ + H]^+^. The same cluster has already been described in the bark, confirming the presence of these compounds in the plant [17]. Two further nodes linked to this compound (asperphenamate) were detected with *m*/*z* 529.208 [C_32_H_30_N_2_O_4_ + Na]^+^ in the ethanolic extract and *m*/*z* 529.21 [C_32_H_30_N_2_O_4_ + Na]^+^ in the methanolic extract. An unpaired *m*/*z* 467.204 node is also observed, exclusively present in the methanolic crude extract. Cluster D analysis showed that two ion pairs (nodes) matched the GNPS (The Global Natural Product Social Molecular Networking) databases. These two MS^2^ spectra have never been described in the genus *Erismadelphus*. The fact that these compounds are present in both types of extractions somehow indicates an almost certain identification of the fragments *m*/*z* 336.222 [C_17_H_14_O_6_ + Na]^+^ and *m*/*z* 334.202 [C_14_H_14_Cl_2_O_5_ + H]^+^, annotated as salvianolic acid F and 3-(3,3-dichloro-2-hydroxypropyl)-6,8-dimethoxyisochromen-1-one, respectively (Figure 2). Salvianolic acids are known not only for their antioxidant activities but also for their efficacy against fibrosis, cancers, and cardiovascular diseases [21,22]. Cluster D also reveals other fragments, notably *m*/*z* 352.217 [C_16_H_31_O_8_ + H]^+^ and *m*/*z* 350.199 [C_16_H_29_O_8_ + H]^+^, corresponding, respectively, to peaks observed at retention times RT = 3.37 min and RT = 3.54 min, predominantly present in the crude ethanolic extract. It is important to underline that several compounds annotated at the level of the leaves are from fungi. In fact, chemical studies made on the bark had already shown the presence of several compounds of fungal origin which makes the studies on this plant quite interesting [17].

### 2.3. Antioxidant Activity

The antioxidant activity of the extracts expressed as TE/g DW (Trolox equivalent per gram dry weight) of the extracts were obtained from the Trolox calibration curve. As previously described in the bark section [17], the antioxidant activity of ethanolic extract of *Erismadelphus exsul* leaves was evaluated with DPPH and FRAP methods using Trolox as the standard and ascorbic acid for comparison. The results in Table 5 show that the crude extract has good iron reduction capacity; its antioxidant activity is better than ascorbic acid for the FRAP test. This high antioxidant activity of the crude extract may be due to the presence of several antioxidant compounds that act synergistically to enhance overall antioxidant activity. This synergy may enhance the reducing effect on ferric ions, making the extract more effective than ascorbic acid, which is an individual antioxidant. On the other hand, its free radical scavenging capacity is low (DPPH test). This means that the compounds present in the extract form a metal complex quite easily, one of the reasons could be the presence of specific structures such as those of flavonoids, known as antioxidants, that favor electron transfer (FRAP test) rather than hydrogen transfer (DPPH test) [23]. These results reinforce those obtained in the bark [17]. Indeed, it was shown in the bark that the plant reacted better to the FRAP test than to the DPPH test, which is in agreement with its use in traditional medicine. Indeed, like the bark, the leaves of *Erismadelphus exsul* are used in the form of a tea, thus facilitating the assimilation of the metabolites in the bloodstream where the iron is found. Compared to the bark, the leaves clearly exhibit less antioxidant activity (2-to-4-times less antioxidant activity). This difference in activity is partly explained by the probable interference of alkaloids present in the bark [17].

### 2.4. Identification of Antioxidant Molecules in Ethanolic Crude Extract

**Figure 3:**
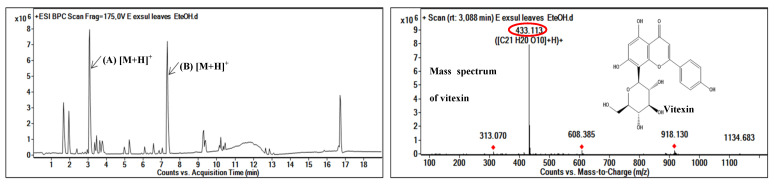
HPLC-UV-MS analyses of the ethanolic crude extract of *Erismadelphus exsul* leaves and the mass spectrum of vitexin.

The chromatogram obtained from the ethanolic crude extract shows two major peaks, among which are an unknown compound (B) and vitexin (A) (Figure 3); the latter is known for its strong antioxidant activities and better reactivity to the FRAP assay compared to the DPPH assay [24]. In order to link the antioxidant activity of the crude extract to the compounds detected via the molecular network, we examined all the compounds of the flavonoid and isoflavonoids class supposed to be responsible for the antioxidant activity of the ethanolic leaf extract of *Erismadelphus exsul*. In total, six compounds were of particular interest to us among which were the following: (i) *m*/*z* 449.106 (C_21_H_20_O_11_^+^, isoorientin); (ii) *m*/*z* 433.113 (C_21_H_20_O_10_^+^, vitexin); (iii) *m*/*z* 593.1861 (C_28_H_32_O_14_^+^, fortunellin); (iv) *m*/*z* 269.637 (C_16_H_12_O_4_^+^, formononetin); (v) *m*/*z* 255.071 (C_15_H_10_O_4_^+^, 5,3′-dihydroxyflavone); and (vi) *m*/*z* 283.102 (C_17_H_14_O_4_^+^, 7-methoxy-3-(4-methoxyphenyl-4H-chromen-4-one). Among these compounds, we can notice that only vitexin has been identified with its mass spectrum in the ethanolic extract. We can therefore hypothesize that vitexin is partly responsible for the antioxidant activity of the crude ethanolic leaf extract of the plant *Erismadelphus exsul*.

### 2.5. Antimicrobial Activity

An evaluation of the antibacterial activity of the crude extracts and fractions of *Erismadelphus exsul* bark and leaves was performed. For this purpose, disk diffusion (determination of inhibition zone diameters IZD) and microdilution (determination of minimum inhibitory concentration MIC and bactericidal concentration MBC) methods were used. Standard antibiotics such as ticarcillin, gentamycin, and tetracycline were used as controls with inhibition diameters ranging from 10 ± 0.00 to 30 ± 0.00 mm. The antimicrobial activity of all fractions was compared to controls and crude extract with the objective of targeting the active fractions. At a concentration of 50 mg/mL, Table 6 shows the different diameters of inhibition obtained; the very first interpretation to be made is that the crude extract and fraction 5 of the leaves gave the best results with relatively higher antimicrobial activity. A similar result can be observed in the bark with the crude extract and fraction 4. The F1 bark fraction was active only on the *Salmonella enterica* strain (IZD 24.67 ± 0.47) and F1 leaf on almost all strains except *E. coli* ATCC 25922. This selective activity of the F1 fractions (leaves and bark) suggests that these fractions contain specific molecules to which these strains are sensitive or insensitive. The F4 bark fraction showed the highest inhibition diameters on *Klebsiella pneumoniae* MDR (IZD 22.67 ± 0.47), *E. coli ATCC* 25922 (IZD 20 ± 1.41), and *E. coli ATCC* 8739 (IZD 18 ± 0.94). Among the leaf fractions, F5 and F1 recorded higher IZD on *ESBL E. coli* (27.67 ± 0.47) and MDR *Klebsiella pneumoniae* strains (22.33 ± 0.47), respectively. Compared with the results obtained with the leaves, the bark was slightly more active on most bacterial strains. This may be explained in part by the fact that the antibacterial compounds in *Erismadelphus exsul* are more concentrated in the bark. In order to further our analysis and to determine the mode of action (bactericidal or bacteriostatic) of the tested samples, the MBC/MIC ratios were calculated and the results obtained were interpreted as follows: when the MBC/MIC ratio is between 1 and 2, the crude extract or fraction is bactericidal, and when this ratio is between 4 and 16, the extract is considered as being bacteriostatic [25]. Based on this principle, the results in Table 7 show that the F4 bark and F5 leaf fractions were more active on most strains, with MBC/MIC ratios ranging from 0.62 to 2.5. In fact, the F5 leaf fraction was bactericidal on all strains, and the F4 fraction was bactericidal on most strains, with the exception of *E. coli* ESBL, where it was bacteriostatic. Thus, analysis and interpretation of these results indicate that most antibacterial compounds in *Erismadelphus exsul* (leaves and bark) are mainly eluted in the F4 and F5 fractions.

### 2.6. Cytotoxicity of Crude Leaf and Bark Extracts of Erismadelphus exsul

**Figure 4:**
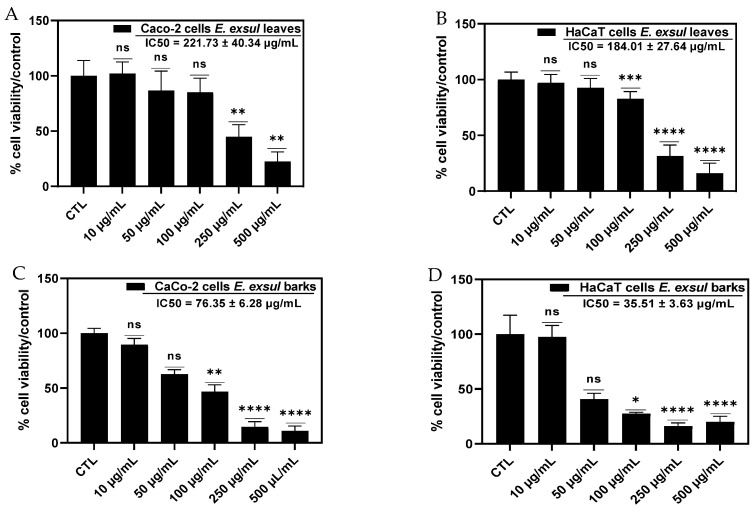
The cytotoxicity of *Erismadelphus exsul* bark and leaves on HaCaT and Caco-2 cells. Differences were observed in comparison with the control (CTL). (**A**) IC50 = 221.73 ± 40.34 µg/mL; (**B**) IC50 = 184.01 ± 24.64 µg/mL; (**C**) IC50 = of 76.35 ± 6.28 µg/mL; (**D**) IC50 = 35.51 ± 3.63 µg/mL. * *p* = 0.0304, ** *p* = 0.0037, *** *p* = 0.0006, and **** *p* < 0.0001 were considered statistically significant and ns was considered insignificant.

Cytotoxicity tests allow for the determination of whether the tested sample inhibits the overall growth of cancer cells and healthy cells. For this purpose, depending on the traditional use of each plant organ (leaves and bark), two cell lines were selected. In the context of using the leaves as an ointment for microbial skin diseases, it was imperative to test its activity on healthy human keratinocyte cells (HaCaT, epidermal cells). We also chose human colon cancer epithelial cells (Caco-2) to test the antiproliferative activity of the bark given its use as a tea. Cytotoxic activity analysis was performed at different extract concentrations ranging from 10 to 500 µg/mL. The IC50 value is determined from the percentage of cell viability compared to the control. The results show that the leaf extract has no cytotoxic activity on healthy HaCaT cells after 24 h of exposure (IC50 184.01 ± 24.64 µg/mL) (Figure 4B). However, bark showed moderate cytotoxic activity on colon cancer cells with an IC50 of 76.35 ± 6.28 µg/mL (Figure 4C). The results obtained in the cytotoxicity study are in line with the plant’s traditional use. Indeed, the leaves of this plant are traditionally applied directly to the skin in the form of an ointment. The absence of cytotoxicity observed on healthy skin cells reinforces the reliability of this traditional use, confirming that topical application of the plant is safe for the skin. In view of these rather interesting results, it was necessary to understand the behavior of each part of the plant on the two cell lines. Thus, we evaluated the cytotoxic activity of the bark on healthy HaCaT cells and that of leaves on Caco-2 colon cancer cells. The results obtained show that leaves have no cytotoxic activity on colon cancer cells (IC50 221.73 ± 40.34 µg/mL) (Figure 4A) while the bark significantly inhibits the proliferation of epidermal cells with an IC50 35.51 ± 3.63 µg/mL (Figure 4D). These results suggest that some compounds present in the bark exert cytotoxic activity against epidermal cells [17]. By making a comparative study of the main families of molecules present in the two parts of the plant, only the cyclopeptide alkaloids and quinolines are absent in the leaves [17]. From this observation, we can easily understand that the moderate antiproliferative activity of the plant is due to the presence of these alkaloids in the bark [17]. The results obtained in the cytotoxicity study are in accordance with the traditional use of the plant. However, as this study was carried out on two cell lines, we cannot conclude that it has cytotoxic activity, as the NCI (National Institute of Cancer) in the United States recommends that cytotoxicity tests be carried out on about sixty cell lines before declaring an extract or the tested compounds to be active [26].

## 3. Discussion

Plants produce secondary metabolites that have specific functions such as protection of the plant against pathogens, allelopathy, and plant–microbe symbiosis at the root level and can also serve as an attractant for pollinators. However, these compounds have several biological properties that are of interest to researchers, especially in the discovery of new molecules that are active against several pathologies in human, animal, and plant health. The primary objective of this work was to identify the plants used by the Fang populations in northern Gabon for the treatment of microbial diseases, with the aim of developing and scientifically validating the use of these plants by the local populations. Therefore, an ethnopharmacological survey was undertaken with about twenty traditional practitioners. Indeed, the selection of plants on the basis of ethnopharmacological data increases the percentage of resulting in a much more active extract. Our field study resulted in the collection of 21 plants, of which 17 were identified. Among the identified plants, *Erismadelphus exsul* Mildbr was selected for phytochemical studies. The choice of this plant was based not only on the limited number of studies but also on the frequency of citation of the plant during the surveys. The second objective was to characterize in a more targeted way the compounds present in the leaf part of the plant. To achieve this, a combined approach of LC-MS/MS mass spectrometry and molecular networks was used. The first step was the extraction of the bioactive compounds from the leaves. For this crucial extraction step, the ideal was to directly recover the traditional extract or to work under the same conditions as the traditional practitioners in order to have approximately comparable results in terms of biological activities. Given the absence of solvents commonly used in the preparation of traditional recipes (palm wine, sugarcane wine, palm oil, and whisky), we opted for an alcoholic extraction based on ethanol and methanol, due to their recognized effectiveness in extracting a wide range of bioactive compounds. Once the choice of solvent had been made, maceration (cold extraction) was chosen as the extraction method. The advantage of this method is that it limits the loss or alteration of fragile organic chemical species, which can degrade at high temperatures. Indeed, depending on the type of extraction and the polarity of the solvent, the variability of the active ingredients can be modified, as can their pharmacological activity. From the leaves of *Erismadelphus exsul*, about twenty compounds were obtained, thus completing the work of Essono Mintsa et al. (2022), who previously identified 18 compounds in the bark, generally belonging to families of molecules such as cyclopeptide alkaloids and quinolines, polyphenols such as flavonoids, and isoflavoinoids, glycerophospholipids, and steroids [17]. Molecular networks designed on the basis of MS/MS spectra not only enabled putative identification but also provided visibility on the main molecular families present in the leaf part, namely (i) flavonoids, (ii) isoflavoinoids, (iii) lignans, (iv) steroids, (v) oligopeptides, (vi) coumarins, and vii) tryptophan alkaloids. Taking into account the results obtained by Essono Mintsa et al. (2022) on the chemical part of the barks, a certain homogeneity was observed in the two types of organs. The main difference in terms of families of molecules between the two types of organs (bark and leaves) is the absence of cyclopeptide alkaloids and quinolines in the leaf part [17]. In fact, only these two families of molecules were not found in the leaves. No concrete explanation has been found for this major difference between the two organs. Nevertheless, some studies have shown that alkaloids are produced as secondary metabolites in response to environmental stress or to provide a defense mechanism against disease-causing aggressions in plants. The third objective of this work was to test the antibacterial and antioxidant activities and to evaluate the cytotoxicity of the leaves and bark of *Erismadelphus exsul* in view of its traditional uses. The results of the antibacterial activities show a high activity of the ethanolic fractions F5 (leaf) and F4 (bark). Based on previous works, it was already demonstrated that the bark of *Erismadelphus exsul* possesses high antimicrobial activity and that compounds such as Mauritine A, Mauritine F, Mauritine A *N*-Oxide, 8-Deoxoantidesmone, 8-Dihydroantidesmone, and Antidesmone are partly responsible for the antimicrobial activity of the bark of *Erismadelphus exsul* [17]. By analogy, we can say that the antibacterial activity observed for the F4 bark fraction is partly due to the presence of these compounds. However, a more targeted mass spectrometry analysis would be important to confirm this hypothesis. In addition, the crude extract and the F5 leaf fraction were bactericidal on the majority of the strains; in view of the chemical groups present in the leaf part, we can assume that the presence of families of molecules such as polyphenols and alkaloids may be responsible for this activity. The evaluation of the antioxidant activity of the leaves shows that the plant reacts better to the FRAP test than to the DPPH test. This means that the compounds present in the extracts and fractions of *Erismadelphus exsul* quite easily form a metal complex. Indeed, the presence of specific structures such as those of flavonoids favor electron transfer (FRAP test) rather than hydrogen transfer (DPPH test). Numerous studies in the literature show that phenolic compounds such as flavones, flavonoids, and isoflavonoids have a great capacity to scavenge free radicals and chelate transition metals such as iron [27]. The high reactivity of *Erismadelphus exsul* leaves in the FRAP assay may be related to the presence of several flavonoids and isoflavonoids. Considering the antioxidant results of the bark part of *Erismadelphus exsul*, we can say that the bark has much more antioxidant activity than the leaf [17]. In this study, it was also a question of proving the safety of this plant. Thus, a study of cytotoxicity was conducted both in the leaf part and in the bark. The results obtained show that the leaves of *Erismadelphus exsul* do not present any cytotoxicity for healthy cells of the skin. These results are in agreement with the traditional use of the leaves. Indeed, the leaves of *Erismadelphus exsul* are used as ointment in the case of skin diseases such as mycosis. In contrast to the leaves, the bark inhibits not only the proliferation of colon cancer cells but also human keratinocyte cells.

## 4. Materials and Methods

### 4.1. Study Area

**Figure 5:**
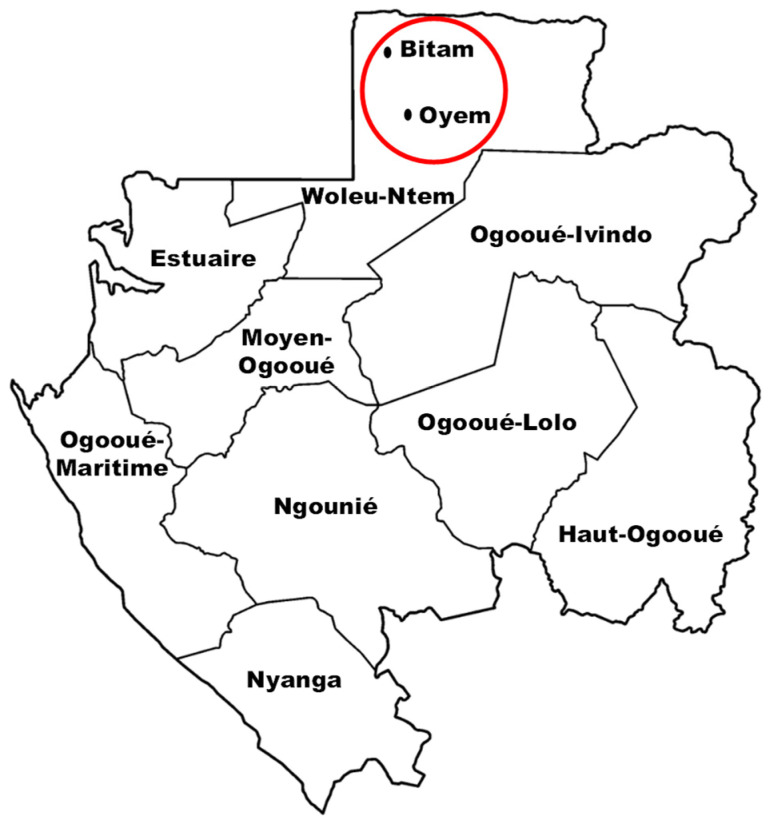
Map of Gabon showing the location of study area Woleu-Ntem. The red circle represents the cities where the surveys were carried out (Oyem, Bitam).

Gabon is a small Central African country marked by a forest that covers 85% (nearly 230,000 km^2^) of its national territory [28]. It is bordered to the northeast by Cameroon, to the northwest by Equatorial Guinea, and to the east by Congo-Brazzaville. Gabon is part of the Congo Basin, the second largest forest in the world after the Amazon. At present, the Gabonese forest is probably the richest in plant species with nearly 20% of the endemic species [29]. Gabon has 13 national parks and 9 provinces covering an area of 267,667 km^2^. This study took place in the province of Woleu-Ntem (northern Gabon) precisely in the cities of Oyem, the provincial capital, and Bitam (Figure 5).

### 4.2. Ethnopharmacological Survey

Ethnopharmacological surveys were conducted between August 2018 and January 2019 in villages located 32 Km from Bitam (Nkoum adzap, Mvane essabeigne, Kone-essong, Nkine, Abem, and Bilé’e-bidoua) and between 15 and 30 km from Oyem (Akam essatouk, Oveng, Adzap be endeng nkodjè, and Akok be essono-obono nkodjè). Information was collected from about 20 traditional practitioners who agreed to share their knowledge of antimicrobial plants and related diseases. The interviewees were identified through the local leaders of the traditional healers committee in said province. The interviewers completed a pre-designed questionnaire. Our interview guide included questions about the local name of the plant, the organ used, the method of preparation, the disease treated, and the age and education level of the informant. It is important to emphasize the fact that healers focus much more on the symptoms, such as fever, cough, and diarrhea, which are a sometimes-direct result of the disease, and not the biological etiology such as the infectious agent responsible for the disease. The survey was conducted in the vernacular Fang language, respecting the traditions and customs of local populations and traditional practitioners.

### 4.3. Collection and Species Identification

Fresh plant samples were collected with secateurs and were labeled and transported in a UV-resistant plastic bag. An herbarium sheet was made where the plants were collected, and plant identification was carried out at Gabon’s national herbarium IPHAMETRA (Institut de Pharmacopée et de Médecine Traditionnelle). An agreement with the focal point of the convention on biological diversity was signed before the work began.

### 4.4. Quantitative Analysis of Ethnopharmacological Data

Descriptive statistical methods were used to analyze ethnopharmacological survey data and quantitative indices, including the informant consensus factor (ICF) and relative citation frequency (RCF). Data were presented as proportions and percentages. ICF and RFC values were calculated using the following formulas: ICF = [Nur−Nt]/[Nur−1].

Where “Nur” is the number of use reports for a particular disease category and “Nt” indicates the number of taxa used for a particular disease category [30,31]. The range of ICF values is between 0 and 1 (0 < ICF < 1) [32].

RCF=CFN Or Citation Frequency (CF)=Number of citations of a particular speciesTotal number of citations of all species×100

“N” is the total number of informants contributing to the survey [33]. The range of RCF values is between 0 and 1 (0 < RCF < 1).

### 4.5. Extraction and Fractionation

The extraction protocol for *Erismadelphus exsul* bark and leaves was performed according to the method used by Essono Mintsa et al., 2023 [25]. After drying and grinding, 400 g of powder were subjected to stirred maceration with 1500 mL of 70% ethanol for 24 h. The extract obtained was filtered under vacuum and concentrated using a rotary evaporator to produce crude extracts. These extracts were then fractionated via flash chromatography on an 80 g Reveleris^®^ Grace Silica PF-50SIHC-F0080 cartridge (Thermo Fisher Scientific, Rheinland-Pfalz, Germany) using a CH_2_Cl_2_ gradient with increasing proportions of MeOH (100:0 to 0:100) at a fixed flow rate of 60 mL/min. The fractions obtained were pooled via thin-layer chromatography (TLC) and analyzed via HPLC-QTOF-HRES to identify potential new compounds.

### 4.6. Data-Dependent LC-HR-ESI-MS^2^ Analysis

LC-HR-ESI-MS^2^ analyses and molecular networking were carried out in accordance with methods described previously by Essono Mintsa et al., 2022 [17]. Samples were analyzed on an Agilent 6530 mass spectrometer (Agilent, Santa Clara, CA, USA) with an ESI source in positive ion mode, coupled to high-performance liquid chromatography on a Sunfire^®^ C18 column (Waters Corp, Milford, MA, USA) (150 × 2.1 mm, 3.5 µm). A standard elution gradient with H_2_O and MeOH mobile phases, containing 0.1% formic acid, was applied at a flow rate of 0.25 mL/min, ranging from 5:95 to 100:0 (MeOH + 0.1% HCOOH) over 40 min. LC-UV and MS data were processed with MassHunter Workstation software 11.0, enabling rapid identification of the chemical components present in the samples and contributing to understanding the therapeutic properties of the extracts studied. 

### 4.7. Molecular Networking Parameters

Molecular networking was performed according to a protocol already described by Essono Mintsa et al. (2022) [17]. Conversion of MS^2^ data from .d format (Agilent Technologies, Massy, France) to .mzXML format with MSConvert is the first step in molecular networking. Next, the .mzXML files are processed with MZmine 2 v32 for mass detection, using a noise level of 3.0 E3 and specific parameters for the ADAP chromatogram builder and ADAP wavelet deconvolution algorithm. MS^2^ scans are matched to strict *m*/*z* and RT tolerances, and isotopes are grouped to defined tolerances. The join alignment module aligns peaks to *m*/*z* and RT tolerances. The .mgf spectral data files and .csv metadata are exported to GNPS-FBMN to generate the molecular network, with MS/MS mass and fragment ion tolerances of 0.02 Da. Molecular array data are then analyzed and visualized using Cytoscape software (version 3.9.0).

### 4.8. Antioxidant Activity: FRAP and DPPH Assays

The Ferric Reducing Power (FRAP) and DPPH assays were carried out in accordance with methods described previously by Essono Mintsa et al., 2022 [17]. The FRAP reagent was prepared by mixing 300 mM acetate buffer pH 3.6, 10 mM TPTZ solution, and 20 mM FeCl3 in a 10:1:1 ratio just prior to use [17]. Trolox (Sigma-Aldrich, St. Louis, MI, USA) was used as a standard. From a 750 µM stock solution of Trolox prepared in ethanol, serial dilutions were made to obtain a concentration ranging from 50 µM to 375 µM. Each standard or sample dilution (50 µL) was combined with 950 µL of FRAP solution. Each analysis was performed in triplicate, then incubated at 37 °C for 30 min. Absorbances were read at 593 nm using a UV spectrophotometer (Anthelie Advanced, Seconam, Shimadzu, France).

### 4.9. Bacterial Germs Tested

The bacteria used (*Escherichia coli* ATCC 25922, *Escherichia coli* ATCC 8739, *Klebsiella pneumoniae* MDR, *Escherichia coli* ESBL, and *Salmonella enterica*) in this study were obtained at the Centre Interdisciplinaire de Recherches Médicales de Franceville (CIRMF) in Gabon.

### 4.10. Antibacterial Test

The sensitivity of microorganisms to plant extracts was studied using the diffusion method [25]. From the bacterial colonies, an inoculum was prepared at a density equivalent to 0.5 McFarland on Muller-Hinton Agar medium (Liofilchem, Roseto, Italy). After inoculation of the bacterial suspension, the agar was dried for 10 min. Then, sterile Wattman paper discs impregnated with 20 µL of extract prepared at a concentration of 100 µg/mL (diluted with 1% DMSO) were placed in the Petri dishes and incubated for 18–24 h at 36 °C. Reference antibiotics such as ticarcillin, tetracycline, and gentamicin were used as positive controls, and each test was performed in triplicate. Minimum inhibitory concentration (MICs) and bactericidal concentration (MBCs) were determined using the microdilution method on sterile 96-well microplates [34]. Series of seven dilutions of each extract (twofold dilution ranging from 0.0049 to 5 mg/mL) were performed in Muller–Hinton nutrient broth (Liofilchem, Roseto, Italy). In order to determine the MBC, the nutrient agar was inoculated with 100 µL of the well contents. After 24 h of incubation at 37 °C, the MBC was determined.

### 4.11. Cell Culture

The cell lines used in this study were selected based on the traditional uses of the two plant organs (leaves and bark); they are human colon epithelial cells, Caco-2 (accession number 86010202, European collection of authenticated cell cultures (ECACC), Salisbury, UK), and immortalized human keratinocyte cells, HaCaT (accession number 300493, Cell Lines Service GmbH, Eppelheim, Germany) [25]. 

### 4.12. CCK-8 Cell Viability Assays

The Cell Counting Kit-8 (CCK-8) assay (Dojindo Laboratories, Kumamoto, Japan) was used to determine cell viability according to the previously described protocol by Essono Mintsa et al. (2023) [25]. The percentage of live cells in DMSO was used to calculate cell viability, using GraphPad Prism 9.3.0 software by applying the following formula:

$$(\%)\;\mathrm{Cell}\;\mathrm{viability}=\frac{Mean\;OD\;of\;treated\;cells}{Mean\;OD\;of\;DMSO\;control\;cells}\times 100\;$$

Data were expressed as mean ± standard deviation (SD) of independent triplicate experiments (N = 3), and results were analyzed using a one-way analysis of variance (ANOVA), the Kruskal–Wallis test, and Dunn’s post hoc test.

## 5. Conclusions

The ethnopharmacological survey carried out among traditional practitioners made it possible to collect 21 medicinal plants used by the Fang populations of northern Gabon in the treatment of microbial diseases. This survey revealed the different parts of the plants used in the preparation of medicinal recipes for microbial control. Thus, through a combined approach of mass spectrometry and molecular networks, 19 compounds were identified. The results of the antibacterial activity analyses for the plant show that, apart from the crude extracts, the fractions F4 (bark) and F5 (leaf) have the best antimicrobial activity. This study also shows that the presence of families of molecules such as polyphenols of flavonoids and isoflavonoids provide the plant with good antioxidant activity. Cytotoxicity studies of *Erismadelphus exsul* bark and leaves show that the leaf does not show any cytotoxicity towards the two cell lines (Caco-2 and HaCaT), and that the bark shows a moderate cytotoxicity on both cell types. The results obtained in this study are rather encouraging, considering the traditional use of the plant. These results provide preliminary indications of the plant’s therapeutic potential. However, to gain a full understanding, further research is required. In particular, more advanced studies, including in vivo tests that take into account compound bioavailability and metabolism, are essential. In addition, it would be useful to examine in detail the phytochemical composition of each plant collected in this survey to gain greater insight into their therapeutic efficacy.

## Figures and Tables

**Table 1:** Socio-demographic data of traditional healers.

Variable	Categories	Number of Traditional Practitioners	Percentage (%)
Level of Education	Uneducated	2	10
	Primary school	3	15
	High school	7	35
	College	3	15
	Postgraduate qualification	5	25
Age (years)	<20	3	15
	(20–40)	2	10
	(40–50)	12	60
	(60–70)	3	15
	>30	1	5
Experience (years)	>20	5	25
	<10	14	70
Gender	Male	12	60
	Female	8	40

**Table 2:** Disease categories based on the information consensus factor (ICF).

Categories	Nur	Nt	ICF
I Diseases associated with infections	18	12	0.35
II Diseases associated with skin	13	3	0.83
III Diseases related to digestive problems	7	6	0.16
IV Diseases associated with fever and coughs	9	5	0.50
V Diseases associated with infertility	6	4	0.40

Nur: Number of use reports for particular disease category; Nt: Number of taxa used for a particular disease category.

**Table 3:** Antimicrobial plants used by the Fang populations of northern Gabon.

Species	Family	Vernacular Names ^a^	Parts Used	Recipes Preparation	Mode of Administration	Indication of Disease Treated	RCF
*Erismadelphus exsul* Mildbr	Vochysiaceae	Essang afane	BL	Decoction	Oral and dermal route	Urinary tract infections, pneumoniae, bronchitis, and sexually transmitted diseases	0.59
*Baillonella toxisperma* Pierre	Sapotaceae	Adzap	BL	Decoction	Oral route	Fever, gastrointestinal pain, and urinary diseases, rheumatic pains, mycosis, ringworms, wounds, and malaria	0.39
*Morinda lucida* Benth	Rubiaceae	Akeng	BL	Decoction	Oral route	Internal hemorrhoids and pulmonary infections	0.25
*Petersianthus macrocarpus* P. Beauv	Lecythidaceae	Abing	BL	Decoction	Oral route	Cough, fever, and sexually transmitted diseases	0.15
*Entandrophragma cylindricum* (Sprague) Sprague	Meliaceae	Assiée	BL	Decoction	Oral route	Stomach pain and abdominal pain	0.05
*Ongokea gore* (Hua) Pierre	Olacaceae	Angeukk	B	Decoction	Oral route	Constipation	0.25
*Trichoscypha acuminate* Engl.	Anacardiaceae	Amvout	BL	Decoction	Oral and rectal route	Male infertility and dysentery	0.25
*Pentaclethra macrophylla* Benth.	Mimosaceae	Ebeigne	B	Decoction	Oral route	Diarrhea	0.10
Unidentified	Unidentified	Mebamane Elé’e	B	Decoction	Oral route	Anti-poison and fever	0.40
*Omphalocarpum elatum* Miers	Sapotaceae	Mebeignemegône	B	Maceration	Oral route	Male infertility and chronic diarrhea	0.40
*Pycnanthus angolesis* (Welw.) Warb.	Myristicaceae	Eteng	B	Infusion	Oral route	Powerful antibiotic against sexual diseases and gonorrhea	0.10
*Erythrophleum ivorense* A. Chev	Fabaceae	Elone	B	Decoction	Dermal and oral route	Eczema, burns, and ulcers	0.45
Unidentified	Unidentified	Ngône	B	Maceration	Oral and dermal route	Eczema, wounds, and gonorrhea	0.15
*Parkia bicolor* A. Chev	Mimosaceae	Essang	B	Infusion	Rectal and oral route	Hemorrhoids and chronic coughs	0.10
*Chlorophora exceisa* (Welw) Benth.	Moraceae	Abang Elé’e	B	Maceration	Oral route	Fever and diarrhea	0.20
*Guibourtia tessmannii* (Harms) J. Leonard	Fabaceae	Oveng	BL	Infusion	Oral route	Sexual weaknesses and sexually transmitted diseases	0.40
Unidentified	Unidentified	Abèe	BL	Powder	Rectal route	Female infertility and fallopian tube obstruction in women	0.15
*Bobgunnia fistuloides* (Harms) J.H. Kirkbr. & Wiersema	Mimosaceae	Akok Elé’e	B	Maceration	Anal route	Hemorrhoids	0.20
Unidentified	Unidentified	Akora Elé’e	BL	Maceration	Oral route	Body and mouth odor due to bacteria	0.10
*Cylicodiscus gabunensis* Harms	Mimosaceae	Edoum	B	Powder	Oral route	Sexual infections	0.25
*Ceiba pentandra* (L.) Gaertn.	Malvaceae	Doum	B	Decoction	Oral route	Clogged trumpets	0.15

Note: BL, the abbreviation of Bark and Leaves; B, the abbreviation of Bark; ^a^, indicates that the language of the investigation is Fang.

**Table 4:** Compounds putatively identified in the molecular network obtained from the crude extract of *Erismadelphus exsul* leaves.

Precursor *m*/*z*	Exact Mass	Molecular Formula	Adduct	Cosine Score	Compound Name	Superclass
433.110	432.106	C_21_H_20_O_10_	[M + H]^+^	0.94	Vitexin	Flavonoids
595.168	594.163	C_27_H_30_O_15_	[M + H]^+^	0.90	Kaempferol 3-neohesperidoside	Flavonoids
665.317	663.647	C_34_H_48_O_13_	[M + H]^+^	0.87	Sarmentoside B	Steroids
269.081	268.640	C_16_H_12_O_4_	[M + H]^+^	0.86	Formononetin	Isoflavonoids
801.445	801.444	C_45_H_57_N_3_O_9_	[M + NH_4_]^+^	0.85	Beauvericin	Oligopeptides
433.222	433.231	C_24_H_32_O_7_	[M + H]^+^	0.83	Grandisin	Lignans
449.108	449.111	C_21_H_20_O_11_	[M + H]^+^	0.82	Isoorientin	Flavonoids
593.274	593.27	C_35_H_36_N_4_O_5_	[M + H]^+^	0.82	Pheophorbide A	Tryptophan alkaloids
445.212	444.205	C_27_H_28_N_2_O_4_	[M + H]^+^	0.81	Asperglaucide	Small peptides
283.091	282.089	C_17_H_14_O_4_	[M + H]^+^	0.81	4′,7-Dimethoxyisoflavone	Isoflavonoids
327.229	304.240	C_20_H_32_O_2_	[M + Na]^+^	0.80	Drostanolone	Steroids
355.202	354.194	C_21_H_26_N_2_O_3_	[M + H]^+^	0.73	Ervatamine	Tryptophan alkaloids
337.060	314.079	C_17_H_14_O_6_	[M + Na]^+^	0.73	Salvianolic acid F	Stilbenoids/Lignans
333.029	334.022	C_14_H_14_Cl_2_O_5_	[M + H]^+^	0.73	3-(3,3-dichloro-2-hydroxypropyl)-6,8-dimethoxyisochromen-1-one	Coumarins
255.065	255.070	C_15_H_10_O_4_	[M + H]^+^	0.70	5,3′-Dihydroxyflavone	Flavonoids
438.228	396.194	C_24_H_28_O_5_	[M + ACN + H]^+^	0.84	(*E*)-3-(4-methoxyphenyl)-1-[2,4,6-trimethoxy-3-(3-methylbut-2-enyl)phenyl]prop-2-en-1-one	Flavonoids
507.019	507.232	C_32_H_30_N_2_O_4_	[M + H]^+^	0.94	Asperphenamate	Small peptides
402.213	403.207	C_25_H_26_N_2_O_3_	[M + H]^+^	0.84	Aurantiamide	Small peptides

**Table 5:** Antioxidant properties of the crude extract (Ce) and fractions (F1–F5) of *Erismadelphus exsul* leaves.

Sample	FRAP (µM TE/g DW)	DPPH (µM TE/g DW)
EE EtOH LCe	1023.09 ± 11.53	40.56 ± 1.31
EE EtOH LF1	200.28 ± 9.15	48.54 ± 2.72
EE EtOH LF2	112.82 ± 4.55	46.18 ± 6.34
EE EtOH LF3	231.15 ± 72.77	180.43 ± 24.03
EE EtOH LF4	510.08 ± 2.65	123.88 ± 39.61
EE EtOH LF5	482.72 ± 31.27	209.05 ± 5.57
Ascorbic Acid	631.99 ± 39.08	1261.79 ± 150.68

The results are expressed as mean ± standard deviation (S.D.); EE = *Erismadelphus exsul* and DW = Dry weight. LCe = Leaves crude extract, F = Fractions, L = Leaves, and TE = Trolox equivalent.

**Table 6:** Inhibition zone diameters (IZD) of crude extract and ethanolic fractions of *Erismadelphus exsul*.

Inhibition Zone Diameter (IZD, mm)					
Sample	Bacterial Strains				
	*E. coli* ATCC 25922	*E. coli* ATCC 8739	*K. p* MDR	*S. enterica*	*E. coli* ESBL
EE EtOH BCe	11.67 ± 0.47	21.33 ± 0.47	12.33 ± 0.47	13.33 ± 0.47	14.33 ± 0.47
EE EtOH BF1	Na	Na	Na	24.67 ± 0.47	Na
EE EtOH BF2	10 ± 0.00	11 ± 0.00	13.33 ± 0.47	12 ± 0.00	9 ± 0.00
EE EtOH BF3	11 ± 0.00	11.33 ± 0.47	9 ± 0.00	9.33 ± 0.47	9.33 ± 0.94
EE EtOH BF4	20 ± 1.41	18 ± 0.94	22.67 ± 0.47	9 ± 0.00	10 ± 0.00
EE EtOH BF5	10 ± 0.82	Na	11.33 ± 0.94	13 ± 0.00	12.67 ± 0.47
EE EtOH LCe	12 ± 0.82	17 ± 0.47	11.33 ± 0.47	17.33 ± 0.47	20.33 ± 0.94
EE EtOH LF1	Na	10 ± 0.00	22.33 ± 0.47	10.33 ± 0.94	20.67 ± 0.44
EE EtOH LF2	13.33 ± 0.47	9.67 ± 0.47	12.33 ± 0.82	9.00 ± 0.00	14.67 ± 0.44
EE EtOH LF3	9.00 ± 0.00	Na	Na	Na	8.00 ± 0.00
EE EtOH LF4	12 ± 0.00	Na	8.33 ± 0.47	Na	Na
EE EtOH LF5	14.33 ± 0.47	17.67 ± 1.70	22 ± 0.00	17.67 ± 0.47	27.67 ± 0.47
Ticarcillin	19.33 ± 0.47	21.33 ± 0.47	20 ± 0.00	20 ± 0.00	Na
Gentamicin	30 ± 0.00	23 ± 0.00	21 ± 0.00	22.33 ± 0.47	22 ± 0.00
Tetracycline	Na	Na	12 ± 0.00	Na	Na
1% DMSO	Na	Na	Na	Na	Na

EE = *Erismadelphus exsul*; BCe = Bark crude extract; LCe = Leaves crude extract; *E. coli* = *Escherichia coli*; *K. p* MDR = *Klebsiella pneumoniae* multi-drug-resistant; *S. enterica* = *Salmonella enterica*; *E. coli* ESBL = *Escherichia coli* extended-spectrum beta-lactamase-producing; EtOH = ethanolic; F = fraction; Na = not active.

**Table 7:** Minimum inhibitory concentrations (MICs) and bactericidal concentrations (MBCs) of crude extract and ethanolic fractions of *Erismadelphus exsul*.

MIC and MBC (mg/mL)										
Sample	*E. coli* ATCC 25922		*E. coli* ATCC 8739		*K. p* MDR		*S. enterica*		*E. coli* ESBL	
	MIC	MBC	MIC	MBC	MIC	MBC	MIC	MBC	MIC	MBC
EE EtOH BCe	1.25	5	1.25	2.5	0.62	5	0.62	5	1.25	2.5
EE EtOH BF1	Nt	Nt	Nt	Nt	Nt	Nt	1.25	1.25	Nt	Nt
EE EtOH BF2	1.25	5	1.25	5	0.62	2.5	0.62	2.5	2.5	5
EE EtOH BF3	2.5	5	2.5	>5	5	>5	2.5	>5	2.5	5
EE EtOH BF4	1.25	2.5	0.62	1.25	1.25	2.5	1.25	2.5	0.62	2.5
EE EtOH BF5	2.5	>5	Nt	Nt	2.5	>5	1.25	2.5	1.25	2.5
EE EtOH LCe	0.62	5	1.25	2.5	0.62	5	1.25	2.5	1.25	2.5
EE EtOH LF1	Nt	Nt	Nt	Nt	1.25	2.5	0.62	5	1.25	1.25
EE EtOH LF2	1.25	2.5	0.62	2.5	2.5	>5	0.31	5	0.62	2.5
EE EtOH LF3	2.5	>5	Nt	Nt	Nt	Nt	Nt	Nt	2.5	>5
EE EtOH LF4	2.5	0.62	Nt	Nt	0.62	5	Nt	Nt	Nt	Nt
EE EtOH LF5	1.25	1.25	1.25	1.25	1.25	1.25	1.25	1.25	1.25	1.25

EE = *Erismadelphus exsul*; BCe = Bark crude extract; LCe = Leaves crude extract; *E. coli* = *Escherichia coli*; *K. p* MDR = *Klebsiella pneumoniae* multi-drug-resistant; *S. enterica* = *Salmonella enterica*; *E. coli* ESBL = *Escherichia coli* extended-spectrum beta-lactamase-producing; EtOH = ethanolic; F = fraction; Nt = not tested.

## Data Availability

Data are contained within the article.
